# Alcoholic Beverage Consumption and Chronic Diseases

**DOI:** 10.3390/ijerph13060522

**Published:** 2016-05-24

**Authors:** Yue Zhou, Jie Zheng, Sha Li, Tong Zhou, Pei Zhang, Hua-Bin Li

**Affiliations:** 1 Guangdong Provincial Key Laboratory of Food, Nutrition and Health, School of Public Health, Sun Yat-Sen University, Guangzhou 510080, China; zhouyue3@mail2.sysu.edu.cn (Y.Z.); zhengj37@mail2.sysu.edu.cn (J.Z.); zwky740359@163.com (T.Z.); daidaolangman@126.com (P.Z.); 2 School of Chinese Medicine, The University of Hong Kong, Sassoon Road, Hong Kong 999077, China; lishasl0308@163.com; 3 South China Sea Bioresource Exploitation and Utilization Collaborative Innovation Center, Sun Yat-Sen University, Guangzhou 510006, China

**Keywords:** alcoholic beverage, wine, cancer, cardiovascular disease, diabetes, obesity

## Abstract

Epidemiological and experimental studies have consistently linked alcoholic beverage consumption with the development of several chronic disorders, such as cancer, cardiovascular diseases, diabetes mellitus and obesity. The impact of drinking is usually dose-dependent, and light to moderate drinking tends to lower risks of certain diseases, while heavy drinking tends to increase the risks. Besides, other factors such as drinking frequency, genetic susceptibility, smoking, diet, and hormone status can modify the association. The amount of ethanol in alcoholic beverages is the determining factor in most cases, and beverage types could also make an influence. This review summarizes recent studies on alcoholic beverage consumption and several chronic diseases, trying to assess the effects of different drinking patterns, beverage types, interaction with other risk factors, and provide mechanistic explanations.

## 1. Introduction

The term alcoholic beverage refers to drinks such as beer, wine or spirits (liquor) containing alcohol. According to the data from WHO, worldwide total *per capita* (15+ years) consumption of pure alcohol was estimated to be 13.5 g/day, and spirits accounted for 50.1% of total consumption of recorded alcoholic beverages, followed by beer (34.8%) and wine (8.0%) [1]. The growing interest in chronic diseases in recent years has led to increasing attention being directed towards drinking, which is one of the most important modifiable factors for many chronic diseases. To fully appreciate how drinking affects disease development, it is helpful to know and understand the different “dimensions” of drinking, *i.e.*, drinking patterns. Drinking patterns include the type of alcoholic beverage as well as amount and frequency of the alcoholic beverage consumption. A standard drink contains 12.5 g of ethanol. Based on drinking quantity and frequency, drinkers can be divided into light, moderate and heavy users corresponding to daily intake of ≤1 drink, 2–3 drinks and ≥4 drinks, respectively [1]. Heavy drinking has been implicated in a wide range of health problems, such as cancer, stroke, ischemic heart disease, diabetes mellitus and obesity [2,3,4,5]. A large percentage of alcohol-related deaths are attributable to cancer, cardiovascular disease and diabetes mellitus [1]. Even when the average daily consumption is low to moderate, heavy episodic drinking (binge drinking), which refers to drinking at least 60 g of pure alcohol on one occasion, has been identified as harmful to humans [6]. In addition to this, other factors, such as diet, gender and age may act as modifiers in the association between drinking and chronic diseases [7]. On the other hand, accumulating evidence has demonstrated the health-promoting effects associated with regular light to moderate red wine consumption [8,9]. Despite the beneficial effects of alcohol itself, non-alcohol components in alcoholic beverage also play a critical role. For instance, the high content of phenolic compounds in red wine, such as anthocyanin, quercetin and resveratrol, has been suggested to be responsible for one of the beneficial effects of drinking [10]. The current review aims to provide information on the association between alcoholic beverage consumption and these chronic disorders. In addition, the health effects of polyphenols in red wine are also discussed. MEDLINE, PubMed, Web of Science, and EMBASE databases were searched for relevant papers published in English in peer-reviewed journals between 2006 and 2016. Studies providing information about association between drinking and selected diseases; or mechanic explanation for the association were included for review. Compared with another recent review of this topic [7], this paper includes obesity, which was absent from the review written by Shield *et al.* In addition, it goes into more depth on certain issues, such as cancer, beverage type, interactions with other risk factors, and the effect of drinking on disease progression [7,11].

## 2. Cancer

According to the International Agency for Research on Cancer, ethanol in alcoholic beverages as well as the acetaldehyde associated with alcohol consumption are classified in Group 1 as “human carcinogens”. Though the carcinogenic ability of ethanol is relatively weak, the high content of ethanol in alcoholic beverages makes it more harmful than many other known carcinogens commonly found in alcoholic beverages [12]. Alcohol consumption has been identified as carcinogenic in a variety of cancers, including those of the upper aero-digestive tract (UADT, including oral cavity, pharynx, larynx, and esophagus), liver, colorectum and female breast (Table 1). It has been well accepted that heavy drinkers have higher risks of total cancer, compared with nondrinkers and occasional drinkers. Relative risks (RRs) for heavy drinkers were 5.13 for oral and pharyngeal cancer, 4.95 for esophageal squamous cell carcinoma, 2.65 for laryngeal, 1.44 for colorectal, 1.61 for breast cancer, all with a clear dose-risk relationship [13]. On the other hand, when it comes to whether light or moderate drinking could increase cancer risks, the evidence is controversial. In addition, it was suggested that consumption of red wine, with its high content of polyphenols and relatively low alcohol concentration, might be protective against certain cancers [14,15,16].

### 2.1. Drinking and UADT Cancers

UADT cancers include malignant tumors of the oral cavity, pharynx, larynx, and esophagus. A meta-analysis reported that the RRs of UADT cancer mortality for drinkers was 2.01 (1.56–2.59) [17]. Furthermore, the risk of UADT cancers increases with increasing consumption of alcoholic beverages [21].

According to a meta-analysis about smoking-betel quid chewing-alcohol interaction effect on oral cancers in Southeast Asia, the pooled OR for drinking was 2.2 (95% CI, 1.6–3.0). Among smoking-drinking-chewing subjects, the individual effects of smoking, drinking and chewing only accounted for 6.7%, 3.1% and 17.7% of the risk, respectively, while the interaction effects were responsible for the remaining [22]. A case-control study in France also reported that the increased risk was only found in heavy drinkers who were also ever smokers and the combined effects were greater than multiplicative ones (>4.5 glasses/day, beer, wine, spirits) [23]. In these situations, alcohol might act more as a solvent and increase the cellular membrane permeability to other carcinogens. For those already diagnosed with oral cancer, reduction (<79 g alcohol/week) or cessation in alcohol drinking resulted in a significant decrease in mortality at three (*p* < 0.001) and five (*p* < 0.001) years [24].

Esophageal cancer (EC) is the eighth most common cancer in the world, causing about 5.4% of all cancer deaths every year according to IARC. It consists of two main histological types: esophageal squamous cell carcinoma (ESCC) and esophageal adenocarcinoma (EAC). ESCC is the dominant type of EC in East Asian countries, and is closely related to dietary factors. Alcohol is an independent risk factor and exerts synergistic effects with other risk factors such as tobacco and salted meat on the development of ESCC. Except for the clinical stage, habitual alcohol drinking was found to be the best predictor of ESCC survival [25]. On the other hand, EAC is more common in Western countries, especially in Caucasian men, with important risk factors such as smoking, gastroesophageal acid reflux and obesity. There was evidence that ORs with drinking years increased for ESCC, whereas for EAC, ORs in drinkers (less than 5 drinks/day) tended to decrease [26]. A cohort study conducted in the UK also reported an inverse association between drinking (≥55.3 g alcohol/week) and the risks of EAC (HR, 0.51; 95% CI, 0.29–0.88), but subgroup analysis based on beverage types showed that the protection was due to wine (HR, 0.49; 95% CI, 0.23–1.04; *p* = 0.06, drinkers *vs.* non-drinkers), rather than beer or spirits. Therefore, other components in wine, instead of alcohol itself, might provide benefits [27]. The regional and race differences of the two histological types indicated the possible role of gene-environment interactions in EC development. Data showed that subjects carrying the GG variant homozygote of alcohol dehydrogenase-2 (ADH2) G48A had a higher EC risks among Asian populations, and heavy drinking made it worse, whereas researchers found that functional genotypes of enzymes involved in alcohol metabolism were not significantly related to EAC or ESCC in a European population [28].

### 2.2. Drinking and Liver Cancer

Alcohol is a well-established risk factor of chronic liver disease and hepatocellular carcinoma (HCC). Previous researches suggested that it was responsible for 32%–45% of HCC. Compared with non-drinking, consumption of more than 3 drinks/day was associated with an increased risk of HCC (HR, 1.92; 95% CI, 1.42–2.60) [29]. The interaction of alcohol and other factors in the development of HCC should also be considered. It was suggested that the deleterious effects of alcohol on one-carbon metabolism could play a role in the development of HCC [29]. A study showed that high folate intake ameliorated the relationship between alcohol consumption and HCC incidence [29]. CYP2E1, a member of the cytochrome P-450 superfamily, can be induced by alcohol and catalyze the formation of reactive oxygen species (ROS). CYP2E1 Pst I/Rsa polymorphism could interact with alcohol consumption and increase the risk of HCC [30]. A prospective study in Taiwan reported that alcohol use and obesity had a synergistic association with the risk of HCC after multivariable adjustment (HR, 3.82; 95% CI, 1.94–7.52) [31].

### 2.3. Drinking and Colorectal Cancer

The relation between quantity of alcohol consumption and colorectal cancer incidence follows a J-shaped curve. Compared with non/occasional drinkers, light to moderate drinking had no significant effect on colorectal cancer risk [32]. A study even suggested that moderate alcohol consumption was associated with decreased risk of colorectal in both men (OR, 0.35; 95% CI, 0.16–0.74) and women (OR, 0.40; 95% CI, 0.18–0.91) [14]. Subgroup analysis indicated the protective effect was related to red wine, but heavy drinkers had significantly increased risks when it came to colorectal cancer. The adverse effect was especially higher for men than for women [32]. It is of note that the association differed by family history of colorectal cancer. Drinkers (≥30 g/day) with a family history of colorectal cancer (RR, 2.02; 95% CI, 1.30–3.13) had higher risks than those with no family history (RR, 1.23; 95% CI, 0.96–1.57) [33]. Another study on the combined effects of XRCC1 polymorphisms and alcohol consumption also supported the role of gene-environment interactions in colorectal cancer susceptibility [34]. It is known that alcohol can interfere with one-carbon metabolism, which is associated with colorectal cancer risk [35]. A prospective study found that folate fortification might attenuate the detrimental effect of heavy alcohol consumption (≥30 g alcohol/day) on colorectal cancer risks [35].

### 2.4. Drinking and Breast Cancer

Although alcohol has been confirmed as a risk factor of breast cancer, risks vary significantly with other factors such as menopausal status, specific tumor characteristics and race, and can be affected by folate intake and hormone therapy. A large cohort study in France reported that in the premenopausal period, no association could be found between high alcohol consumption (>20 g alcohol/day), whatever beverage types (wine, beer/cider, fortified wine and spirits), and increased risks of breast cancer, while for drinkers in the postmenopausal period, a significant linear association was found for breast cancer (*p* < 0.0001) (>20 g alcohol/day, mainly wine and beer) [36]. Data from another prospective research supported the view and suggested that even light to moderate drinking (5–30 g/day of alcohol) could increase breast cancer risk for postmenopausal women [37]. Besides, it was reported that the effect of alcohol on breast cancer was restricted to estrogen receptor-positive (ER^+^) cancer [38]. According to a case control study targeted at African-American women, no association could be found between recent drinking and the risk of breast cancer, and for subjects who drank when under 20 years of age, a decreased risk with lifetime alcohol drinking was found (OR, 0.65; 95% CI, 0.47–0.89) [39]. A case control study in Japan found that a high folate intake ameliorated excess breast cancer risks related to alcohol consumption for postmenopausal women [40]. On the other hand, for postmenopausal women, the hormone therapy seemed to exacerbate the risk of breast cancer with alcohol drinking [41].

In addition to being a risk factor, the consumption of alcohol has been suggested to affect the course of breast cancer. Researchers found a modest but significant association between pre-diagnostic drinking (>2 units/day) and breast cancer recurrence [42]. For those already diagnosed with breast cancer, post-diagnostic alcohol use (≥6.0 g/day) increased the risk of recurrence only for postmenopausal women. However, considering the totality of the evidence, no association was found between post-diagnostic drinking and breast cancer patients’ survival [43].

### 2.5. Drinking and Pancreatic Cancer

Heavy drinking has been widely accepted to be a major cause of chronic pancreatitis and a risk factor of type 2 diabetes mellitus, both of which are associated with pancreatic cancer. Compared with none or occasional drinkers (<1 drink/day), light to moderate drinkers (<4 drinks/day) didn’t show increased risks of pancreatic cancer. However, subjects with a high consumption level (>9 drinks/day) exhibited high risks (OR, 1.6; 95% CI, 1.2–2.2) [44]. Studies also suggested that other predisposing factors besides alcohol were involved in the process. For example, it was reported that smoking could potentially multiply pancreatic risks for drinkers. Alcohol and tobacco consumption was found to be associated with an earlier onset of pancreatic adenocarcinoma, and the detrimental effects could last for 10 years after abstinence [45].

### 2.6. Drinking and Other Cancers

Results from a recent study showed that prenatal alcohol exposure induced histophysiological changes in the prostate and increased the susceptibility of the prostate to neoplasia in the adulthood [46]. According to a case-control study in Alberta, Canada, lifetime alcohol consumption (292.34 kg, lifetime), rather than current intake, was positively associated with both aggressive (OR, 2.00; 95% CI, 1.19–3.36) and non-aggressive (OR, 1.78; 95% CI, 1.19–2.66) prostate cancers [47].

High-risk Human Papillomavirus (HR-HPV) persistence has been confirmed as a major step in cervical carcinogenesis. Prospective studies showed that alcohol consumption was associated with increased risks of cervical HR-HPV persistence [48,49]. Alcohol drinkers also showed higher risks of cervical intraepithelial neoplasia (CIN) 1 compared with abstainers, and high drinking frequency and quantity would even aggravate the issue [50]. It was reported that relatively modest alcohol drinking, whatever beverage types, was inversely associated with endometrial cancer compared with abstinence (multivariable RR, 0.81; 95% CI 0.68–0.96). Women with light alcohol intake (<5 g/day) had a 22% lower endometrial cancer risk [51].

After adjusting for possible confounding factors (smoking, BMI, educational level), both high drinking frequency (2–7 times/week) and quantity (>100.00 g ethanol/week) were associated with increased risks of gastric cancer. The HR of gastric cancer was 2.00 (95% CI, 1.04–3.82) and 1.90 (95% CI, 1.13–3.18), respectively [52].

Though opinions about the association between total alcohol consumption and risks of skin cancer were controversial, evidence agreed on the fact that a higher current intake of certain alcohol beverages (white wine and spirits) increased the risks of both melanoma and non-melanoma skin cancer [53,54]. In addition, a meta-analysis combined the results from 24 studies reported that alcohol intake was associated with a decreased risk of renal cell carcinoma for both men and women and across different alcoholic beverage types [55].

### 2.7. Potential Mechanisms for Protective Effects of Drinking in Cancer Development

Previous studies have linked a regular and moderate consumption of wine, especially red wine, with a decreased risk of cancer initiation and development [15,16]. Both *in vitro* and *in vivo* studies suggested that the chemopreventive effects should be attributed to the presence of polyphenols in wine. Due to the preservation of grape skin during maceration, the concentration of total phenolics in red wine (1378–2681 mg/L gallic acid equivalents) is much higher than that in white wine (<50 mg/L gallic acid equivalents) [16]. Moderate consumption of red wine (e.g., 125 mL) would deliver about 50 mg polyphenols to the gastrointestinal tract [56]. Potential anti-carcinogenic polyphenols in wine include anthocyanin, phenolic acids, flavonoids (catechin, quercetin, naringenin, apigenin, kaempferol, myricetin) and stilbenes (resveratrol) [10].

In culture, red wine inhibited proliferation and decreased clonogenic survival of A549 human lung cancer cells at a low concentration (0.02%), and white wine mediated similar effects but at higher concentrations (0.5%–2%). Besides, control experiments suggested that the content of alcohol or resveratrol in wine could not explain the anti-proliferative activity of wine [16]. Likewise, in another *in vitro* study, low dose of red wine (1%–5%), but not white wine, showed alcohol- and resveratrol-dependent cytotoxicity in several cancer cell lines [57].

Polyphenolic extracts from red wine, at physiologically relevant concentrations, exhibited proliferation-inhibitory effects in various cancer cells. The effects have been linked with induction of apoptosis and cell cycle arrest. In human colon cancer SNU-C4 cells, the treatment of red wine polyphenols (100 ìg/mL) induced apoptosis via up-regulation of caspase-3 activity and modulation of the BCL-2 family (Bax, Bcl-2) [58]. A study removed anthocyanin from the extracts of red wine to better simulate the profile of polyphenols likely to reach colon, and found that the residual extracts were still capable of inhibiting colon adenocarcinoma cell proliferation, partly via apoptosis and partly *via* G_2_/M cell cycle arrest [56]. In two leukemia cell lines (Jurkat, MOLT-4), red wine extracts dose dependently triggered apoptotic cell death and cell cycle arrest (G_0_/G_1_, G_2_/M) [59,60]. Furthermore, the pro-apoptotic effects might be selective. Red wine polyphenols specifically induced apoptosis and G_1_ cell cycle arrest of embryonic carcinoma cell line P19 without affecting the growth of normal cells in the same culture conditions [61]. In human colon-derived CCD-18Co myofibroblast cells, phenolic extracts of red wine concentration dependently decreased mRNA expression of LPS induced inflammatory mediators (NF-κB and cell adhesion molecules). The anti-inflammatory effects of wine polyphenols might play a role in the prevention of colon carcinogenesis, since chronic pro-inflammatory signals and sustained inflammation could result in colorectal cancer [15]. Interfering with intracellular signal transduction might be one of the mechanisms. Red wine inhibited proliferation of A549 and H1299 human non-small lung cancer cells at low concentrations (0.02%). The effect might be correlated with suppression of basal and epidermal growth factor (EGF)-stimulated Akt and Erk signals and induction of total and phosphorylated levels of p53 [16]. Estrogen plays an important role in estrogen-dependent cancers (e.g., breast and endometrium cancer). Data showed that red wine and alcohol free red wine, but not white wine, could markedly reduce aromatase activity, which catalyzes the aromatization of androgen to estrogen [62].

*In vivo* studies also support the anti-cancer activity of red wine polyphenols. Administration of red wine extracts in mice prevented the appearance of colonic aberrant crypt foci [63]. In an nude mouse model transplanted with the ER-MDA-MB231 breast cancer cells, reduced tumor growth, suppression of genes of the NF-κB pathway as well as up-regulation of cell cycle arrest related genes (CDK5RAP1, RBBP8, and SERTAD1) were all observed after the red wine extracts treatment (100 mg/kg body weight equivalent of polyphenols) [64].

Resveratrol has been suggested to be an important bioactive compound responsible for the chemopreventive effects of red wine. However, according to a recent study, for intake of 100 mL of wine, the margin of exposure was 4.1 for ethanol and 459,937 for resveratrol on average, meaning that ethanol is 100,000 times more potent than resveratrol per glass of wine, so resveratrol alone might not be able to counteract the deleterious effects of ethanol. However, wine contains numerous other polyphenols, such as proanthocyanin, catechin and quercetin, which could also counteract the deleterious effects of ethanol. Recent studies suggested that the efficacy of red wine on carcinogenesis might depend more on synergistic interactions between different bioactive polyphenols. For instance, quercetin and resveratrol, two major bioactive compounds in wine, exerted synergistic anti-proliferative effects, while the presence of catechin, inhibited this interaction [63]. Resveratrol can be rapidly converted to piceatannol by cytochrome P450-1A2. A low concentration of piceatannol (50 nM) was reported to upregulate the expression of the proto-oncogene c-Myc, which was dependent on progesterone receptor and estrogen receptor, resulting accelerated proliferation of MCF-7 breast cancer cells, whereas at higher concentration (25–100 μM), it was anticarcinogenic to the same cell line. The results could explain the contradictory reports about the pro- and anti-cancer effects of piceatannol, and why moderate drinking of wine could increase the risk of female breast cancer [65]. In addition, the effects of some classes of polyphenols on the breast cancer risk could be altered by drinking. A cohort study showed that intakes of hydroxybenzoic acids (HR, 2.28; 95% CI, 1.16–4.49), flavonoids (HR, 2.46; 95% CI, 1.23–4.92), anthocyanin (HR, 2.94; 95% CI, 1.32–6.53), catechin (HR, 2.28; 95% CI, 1.19–4.36), and proanthocyanidin (HR, 2.98; 95% CI, 1.40–6.33) were related to increased breast cancer risk for heavy drinkers, whereas for non/light drinkers, the effects of these polyphenols were protective. It might be the result of the interaction between dietary polyphenols and alcohol in enhancement of circulating estrogen levels [66]. Together, these results might explain discordance in different studies.

### 2.8. Potential Mechanisms for Detrimental Effects of Drinking in Cancer Development

For a 10% incidence of health effect, the lower one-side confidence limit of the benchmark dose for ethanol was 700 mg/kg body weight per day. However, due to the relatively high concentration in alcoholic beverages (2%–80% vol.), the situation was reversed in terms of margin of exposure (MOE) (3.1 for one standard drink per day), which made ethanol more harmful in alcoholic beverages [12].

#### 2.8.1. Modulating Sex Hormone Levels

Previous studies suggested that alcohol increased sex hormone levels in humans, which might be involved in its carcinogenesis [67]. The assumed action of alcohol on sex hormones works through different pathways: stimulation of adrenal androgen productions, up-regulation of luteinizing hormone (LH) thereby promoting ovarian production of androgen, induction of aromatases as well as impairment of the metabolism of sex steroid in liver. According to a large cross-sectional study in Europe, alcohol intake had an impact on serum level of sex hormone and sex-hormone binding globulin (SHBG). Pre-menopausal women who consumed more than 25 g/day of alcohol had elevated serum levels of androgen (dehydroepiandrosterone sulphate, androstenedione, and testosterone), both of adrenal and ovarian origin, which suggested a possible effect of alcohol on steroidogenesis in these glands. Besides, a significant positive association with alcohol consumption was found for estrone (40% higher than non-consumers). The increase of serum androgen and estrone in response to alcohol was also observed in post-menopausal women (25 g/day of alcohol), but to a less extent (10%–20%). In addition, an inverse association was reported between alcohol intake and serum SHBG level only in post-menopausal women. Serum estradiol level seemed to have no relation at all with alcohol in both pre- and post- menopausal women [68].

Several studies supported that the action of alcohol on systemic estrogen levels was related to aromatase. In mammary tumor virus (MMTV)-neu mice, alcohol administration promoted tumor development, which was accompanied by increased systemic estrogen levels as well as expression of aromatase and ERα in the tumors [69]. In addition, it was suggested that alcohol caused breast cancer in postmenopausal women by abolishing peroxisome proliferator-activated receptor (PPAR) c-dependent inhibition of aromatase expression in adipocyte, leading to increased estrogen levels [67].

#### 2.8.2. Promoting Age-Related Biological Processes

Data suggested that alcohol abuse might promote biological processes associated with aging, resulting in early onset of aging-related diseases, including cancer at multiple sites. In alcohol-preferring rat model, which was used to mimic alcohol abusers, chronic alcohol intake exacerbated age-related degenerative changes in hepatocytes, including macrofat, megamitochondria and cytoplasmic degenerative bodies [70]. Shortened telomere length (TL) is recognized as a biological marker of aging, because epidemiology evidence has demonstrated that, at the same age, people with shorter telomeres have higher cancer risks. Biological process that accelerates telomere shortening, such as inflammation and oxidative stress, can also be induced by heavy drinking. In alcohol abusers, TL was nearly halved (geometric means 0.42 *vs.* 0.87 relative T/S ratio; *p* < 0.0001) comparing to the control after adjusting for potential confounding factors (age, BMI, current smoking, vegetables, *etc.*). Furthermore, the decrease of TL was linked with increased drink-units/day (*p* trend = 0.003). Heavy drinker (>40 g/day) had shorter TL than those drinking ≤40 g/day (geometric means 0.48 *vs.* 0.61 T/S ratio; *p* = 0.002). Thus, alcohol abuse may be related to a premature aging at the cellular level [71].

#### 2.8.3. Interfering with Folate Metabolism

Folate plays a key role in the synthesis of S-adenosylmethionine, the universal methyl donor, as well as in nucleotide synthesis. Folate depletion could lead to alterations in DNA and RNA methylation, disruption of DNA integrity and DNA repair, therefore promoting carcinogenensis [72]. Approximately 60%–70% of heavy drinkers are folate deficient. One of the possible causes is a reduction in the absorption of folate in the intestinal tract. Ethanol had a concentration-dependent acute inhibitory effect on both ^3^H-folic acid and ^3^H-methotrexate uptake, and the effect was more potent for the latter [73].

#### 2.8.4. Promoting Cancer Cell Invasion and Metastasis

In addition to acting as a risk factor for carcinogenesis, epidemiological studies also demonstrated that alcohol intake was associated with cancer invasion and metastasis, leading to poor prognosis [74]. Although the underlying mechanism is still a matter of debate, several possible ways have been postulated.

Epithelial-mesenchymal transition (EMT) plays an important role in the progression and metastasis of epithelial-derived cancers including breast, esophageal, colorectal and liver cancers. It has been reported that alcohol may worsen cancer outcome by stimulating EMT in cancers. A research supported the notion by showing that alcohol up-regulated the signature EMT marker vimentin and expression of matrix metalloprotease as well as cell migration in breast and colon cancer cells [74].

Metastasis suppressing genes such as Nm23 encode proteins can inhibit the establishment of metastasis without affecting the growth of primary tumor. In human breast cancer T47D cells, treatment of alcohol enhanced the invasive ability of cancer cells via suppression of Nm23 and Nm23 over-expression effectively inhibited alcohol induced cellular invasion [75].

It has been well established that mast cells are highly involved in the carcinogenesis of a wide range of solid tumors such as colorectal cancer. *In vitro* migration assay showed that the treatment of alcohol markedly up-regulated mast cell-mediated migration. Chronic alcohol feeding also enhanced tumor invasion in mice, which was accompanied by increased mast cell numbers and activities [76].

Tumor angiogenesis, the formation of new blood vessels, is a prerequisite for progression of solid tumors. Vascular endothelial growth factor (VEGF) plays an essential role in tumor angiogenesis and affects tumor aggressiveness. Monocyte chemotactic protein-1 (MCP-1) has been well established as a critical angiogenic chemokine. Previous studies showed that alcohol exposure increased the expression of MCP-1 and VEGF. It has also been implicated that the enhanced expression of VEGF and MCP-1 was mediated by NF-κB and ROS. An *in vivo* study demonstrated that alcohol exposure at a modest dose (2% in drinking water, equivalent to about two drinks in humans) promoted growth and metastasis of mammary tumors in C57BL/6 mice, and MCP-1 was critical for this alcohol-stimulated tumor angiogenesis and progression [77]. Besides, in a HCC xenograft mouse model, alcohol (0.2% in drinking water) significantly accelerated tumor growth rate, angiogenesis, metastasis via upregulating the expression of VEGF and MCP-1 [78].

In addition, a recent study suggested that the deleterious impact of alcohol on skin health may function through decreasing the concentration of antioxidants, leading skin susceptible to free radicals induced by ultraviolet light. Data supported the view, and alcohol consumption induced significant decrease in the carotenoid concentration in the skin and the minimal erythema dose [79].

#### 2.8.5. Possible Carcinogenic Mechanisms of Acetaldehyde

Acetaldehyde associated with alcohol consumption has been classified as carcinogenic to humans (IARC Group 1). Apart from ethanol metabolism, the direct content of acetaldehyde in alcoholic beverages provides additional risks related to drinking. Therefore, the acetaldehyde concentrations in different alcoholic beverages might explain some of the variations in cancer risk associated with beverage types. The highest acetaldehyde concentration was generally found in fortified wines (118 ± 120 mg/L), followed by spirits (66 ± 101 mg/L), wine (34 ± 34 mg/L) and beer (9 ± 2 mg/L) [80]. In addition, it was reported that the acetaldehyde concentration in Calvados (an apple brandy from France) was significantly higher than that in other alcoholic beverage studied. The high concentration of acetaldehyde was suggested to be responsible for the increased risk of esophageal cancer associated with Calvados consumption [80].

Previous *in vitro* study showed that the acetaldehyde DNA-adduct Cr-PdG could be formed at acetaldehyde concentration as low as 100 μM [81]. Assuming an equal distribution between the beverage and saliva, the residual acetaldehyde in the saliva after swallowing could be about 2417 μM for fortified wine, 1387 μM for spirits, 734 μM for wine and 195 μM for beer, which supported the idea that the high concentration of acetaldehyde in alcoholic beverages could be carcinogenic. However, the effects of acetaldehyde directly contained in alcoholic beverages were short-term. The type of alcoholic beverages had no effect on the saliva acetaldehyde concentration 2 min after rinsing of the mouth with an alcoholic beverage and 30 min after drinking, while the influence of acetaldehyde from ethanol became the major factor [81].

Following ethanol treatment, the concentration of acetaldehyde in mammary tissue remained significantly higher than that in plasma for several hours. The accumulation effect of acetaldehyde was dose- and time- related and was mostly due to *in situ* metabolism of ethanol. The accumulated acetaldehyde is a good substrate for other enzymes in breast gland, such as aldehyde oxidase and xanthine oxidoreductase. Activation of these enzymes produces ROS. The acetaldehyde accumulation and the subsequent increased oxidative stress after ethanol administration might play a critical role in the promotion of breast cancer [82]. In addition, some components in diet might inhibit activity of aldehyde dehydrogenase, which could elevate toxicity of aldehyde [83,84,85,86].

## 3. Cardiovascular Diseases

Most epidemiological studies have suggested an inverse association between regular light to moderate drinking and risks of cardiovascular diseases, such as coronary heart disease, heart failure, stroke and peripheral vascular disease [87]. However, the increased risks of alcoholic cardiomyopathy (ACM), hypertension, stroke and myocardial infarction (MI) related to heavy drinking, either in the form of chronic alcoholism or occasional binge drinking, counteract these protection [1,6,88].

### 3.1. Drinking and Coronary Artery Disease

Coronary artery disease, also called ischemic heart disease, is a group of diseases including stable angina, unstable angina, myocardial infarction, and sudden coronary death. A meta-analysis suggested that the relationship between average alcohol consumption and risks of ischemic heart disease was clearly J-shaped. Drinkers with average alcohol consumption of <30 g/day without heavy episodic drinking had the lowest risks of ischemic heart disease (RR, 0.64; 95% CI: 0.53–0.71) in relation to life time abstainers. Besides, compared with men, women could benefit more from low alcohol consumption, but the effects would turn to harmful at lower levels of consumption compared with men [89]. In addition, according to the prospective NHLBI Twin Study, higher usual alcohol consumption was correlated with lower mortality from coronary artery disease, independent of genetic and early life environmental factors, and the protection was similar across alcoholic beverage types. In male twins without baseline coronary artery disease, the within-pair adjusted HRs per 10 g increment in alcohol consumption was 0.90 (95% CI: 0.84–0.97) [90]. Besides, a large case-control study combined observations from 52 countries, and reported an inverse association between low levels of alcohol consumption and MI risks (adjusted OR, 0.87; 95% CI, 0.80–0.94), which was moderate and might vary across different countries. On the other hand, an episode of heavy drinking (6 drinks or more) might increase acute MI risks in the next 24 h, especially for the old [6]. According to prospective studies, patients who survived MI might benefit from moderate alcohol consumption due to decreased risks of all-cause and cardiovascular mortality. In addition, for male patients after MI, less impaired cardiac function might potentiate this U-shaped association [91,92].

### 3.2. Drinking and Stroke

A meta-analysis of prospective studies suggested a U-curve association between alcohol intake and stroke risks. Compared with non-drinkers, low alcohol drinkers (<15 g/day) had decreased risks of total stroke (RR, 0.85; 95% CI, 0.75–0.95) and stroke mortality (RR, 0.67; 95% CI, 0.53–0.85). Moderate drinkers showed little or no difference from non-drinkers, while heavy drinking (>30 g/day) elevated risks of total stroke (RR, 1.20; 95% CI, 1.01–1.43) but not stroke mortality. In addition, the study showed no statistically significant impact of drinking on hemorrhagic stroke risks [93].

Besides the quantity of alcohol consumption, it has been reported that other drinking patterns, such as frequency and occasional binge drinking influenced stroke risks. According to a population-based cohort study among eastern Finnish men, participants who consumed alcohol more than 2.5 times per week had the highest stroke risks (RR, 2.44; 95% CI, 1.11–5.40) [94]. It has been suggested that hangover, a result of acute alcohol intoxication was strongly related to binge drinking. Another population-based cohort study found that middle-aged men, who reported at least one hangover per year, had 2.58-fold (95% CI, 1.24–5.36) ischemic stroke risk relative to those who did not [88].

It is equally important to know the influence of alcohol intake on stroke severity and outcome. There was evidence that pre-stroke alcohol consumption had no significant influence on stroke severity as well as short-term or long-term outcomes [95].

### 3.3. Drinking and Atrial Fibrillation

Evidence suggested that alcohol consumption increased incidence of atrial fibrillation. According to a meta-analysis of prospective studies, alcohol intake, even at light level (1 drink/day), tends to increase incidence of atrial fibrillation relative to non-drinking (RR, 1.08; 95% CI, 1.06–1.10) [96]. Furthermore, based on data from a cohort study in Denmark, in patients with atrial fibrillation, high alcohol consumption (>20 drinks/week for male and >13 drinks/week for female) elevated the risk of thromboembolism and death [97].

### 3.4. Drinking and Hypertension

A recent meta-analysis combined 16 prospective studies (33,904 men and 193,752 women) on alcohol consumption and hypertension risks. For male, though lacking statistically significant association, there was a trend toward increased hypertension risks with low to moderate drinking, and heavy drinking significantly elevated hypertension risks (31–40 g/day: RR, 1.77; 95% CI, 1.39–2.26 and >50 g/day: RR, 1.61; 95% CI, 1.38–1.87). For female, a J-shaped association existed between drinking and hypertension. Less than 10 g/day of alcohol intake rendered protective effects, while drinking more than 20 g/day significantly increased hypertension incidence [98]. Similarly, a J-shaped relationship was found between drinking and all-cause mortality among hypertension patients. The nadir (RR, 0.82; 95% CI, 0.76–0.88) was suggested to locate at 8–10 g/day [99]. In addition, difference in effects of drinking on hypertension between men and women might be the result of different drinking patterns. Compared with women, at similar drinking amount, men tend to have more binge drinking episodes. However, if the drinking patterns are the same, it will lead a higher blood alcohol levels for women than men following consumption of equivalent amount of alcohol because women usually have less body water content than men for the same body weight [7].

### 3.5. Drinking and Heart Failure

A long term community based study reported that modest drinking in early middle age might be correlated with lower risks of heart failure. Compared with abstainers, HR for male subjects consuming up to seven drinks (14 g alcohol) per week was 0.80 (95% CI 0.68–0.94), but this protective association was less definite for female (HR 0.84; 95% CI: 0.71–1.00) [100]. In addition, a meta-analysis suggested that light to moderate alcohol consumption was inversely associated with risks of heart failure (RR, 0.85; 95% CI: 0.78–0.93) [101]. In a cohort of healthy Swedish men aged 45 to 79 years, researchers found a U-shaped association between drinking and risks of heart failure, with a nadir locating at 7–14 standard drinks/week (RR, 0.81; 95% CI, 0.69–0.96) [102]. However, for the elderly (≥65 years old) already suffering from chronic heart failure, a cohort study in Italy demonstrated that moderate alcohol consumption (≤250 mL/day) was related to increased long-term mortality [103].

### 3.6. Potential Mechanisms

A large number of experimental and clinical studies have been conducted to shed light on the mechanisms of action of alcohol in cardiovascular diseases.

#### 3.6.1. Potential Mechanisms for Protective Effects in the Development of Cardiovascular Disease

Potential mechanisms of alcohol in protection against cardiovascular diseases have been suggested. Nitric oxide (NO) as an important endogenous vasodilator, plays a key role in regulating blood pressure and protecting against pathological vascular damage. The dysfunction of the NO pathway is involved in the development of many cardiovascular diseases, such as hypertension, atherosclerosis and cerebral hypoperfusion [104]. It was reported that low concentration of alcohol could promote NO release from endothelium through upregulating NO synthase, while high concentration or chronic consumption of alcohol could impair endothelial function through decreasing NO bioavailability [104]. Angiotensin II and angiotensin converting enzyme (ACE) play an important role in the process of hypertension and atherosclerosis. A study found that ACE activity increased with age in senescent rats. Low doses of alcohol (0.4 g/kg/day in rats, which is equivalent to 30 g/day in human) inhibited ACE activity of senescent rats to the level of young control rats [105]. Besides, in the development of coronary atherosclerosis, local macrophage-induced inflammatory response and secretion of cytokines, especially IL-1β, played a key role in this process. Alcohol treatment to cultured human macrophages decreased the secretion of IL-1β through inhibiting NLRP3 and AIM2 inflammasomes. The findings might partially explain the atheroprotection related to moderate drinking [87]. Myocardial ischemia could lead to serious complications such as heart attack, arrhythmia and heart failure. In a swine model of chronic ischemia, alcohol administration promoted angiogenesis, increased capillary and arteriolar density in non-ischemic myocardium. These changes might increase collateral blood flow to prevent further ischemic insult as well as compensate for the ischemic myocardium. Besides, alcohol supplement also ameliorated apoptosis and promoted myocyte survival in both ischemic and normal part of myocardium by modulating the mammalian target of rapamycin (MTOR) signaling pathway. Together, these changes might provide evidence for the cardioprotective effects of drinking (90 mL 50% ethanol/day) [106,107]. In addition, a cardioprotective association between alcohol use and ischaemic heart disease could not be assumed for all drinkers, even at low levels of intake [108]. Previous studies have shown that mental stress has deleterious effects on the cardiovascular system through dysregulated cardiovascular responsiveness. An experimental study among healthy adults reported reduced cardiovascular responsiveness associated with moderate alcohol consumption (<15.8 g alcohol for female and <23.7 g alcohol for male). Compared with non-drinkers, mental stress-induced responses including heart rate, cardiac output, vascular resistance and blood pressure were less prominent in moderate drinkers [109]. The influence of drinking in brain tissue varied with changes of dose. An *in vivo* study reported that chronic administration of low-dose alcohol (1% in drinking water) to mice provided protection against transient focal cerebral ischemia/reperfusion injury, and the upregulation of PPARγ might underlie the neuroprotective effects of alcohol [110].

Many studies suggested that moderate drinking of red wine could provide benefits to the cardiovascular system, which have largely been attributed to the rich content of phenolic compounds [111,112,113,114]. The free-radical scavenging ability, inhibition of lipid peroxidation (lipoproteins, membranes) and modulation of lipid profiles could be involved in the cardioprotective effects of red wine. In healthy volunteers, significant increase of plasma total phenolic concentrations and HDL cholesterol as well as decrease of plasma lipid peroxidation levels were detected after 2 weeks of moderate red wine intake (375 mL/day) [111]. In another intervention study, healthy men received 30 g alcohol/day. Compared with gin, daily intake of red wine decreased plasma malondialdehyde and oxidized LDL levels, indicating the antioxidant activity of wine polyphenols [112]. Endothelial dysfunction is highly involved in many cardiovascular diseases. A variety of favorable effects of red wine on endothelial function have been reported. For example, the improvement of endothelial function by red wine could protect cardiac function in ischemic myocardium [113]. Besides, red wine could suppress proliferation and migration of vascular smooth muscle cell, which is critical for atherogenesis [114]. The lower cardiovascular risks in moderate wine drinkers were suggested to be the result of NO-mediated effects by red wine polyphenols (such as resveratrol, anthocyanidins and procyanidins). For instance, in a clinical trial in men at high cardiovascular risks, daily intake of dealcoholized red wine (272 mL, total phenols: 733 Eq gallic acid) for 4 weeks decreased systolic and diastolic blood pressure through an NO-mediated mechanism [115]. An *in vitro* study also suggested that the endothelial dependent vasorelaxant effect of alcohol free red wine (100 and 300 μg/mL) was correlated with increased NO concentration [116]. The NO-mediated vasorelaxant effects of red wine phenolic extracts were mainly through activating endothelial NO synthase [117]. The cardioprotective ability of many drugs, such as acetylsalicylic acid, is correlated with suppression of platelet aggregation. Resveratrol showed inhibition against aggregation of human platelets [118]. In healthy male volunteers, 3 weeks of moderate consumption of red wine (200 mL/day) at dinner improved several hemorheological parameters, including significantly increase of red blood cell (RBC) deformability at high shear stress and hematocrit/whole blood viscosity ratio (indication of oxygen carrying capacity), as well as decrease of RBC aggregation. These changes might contribute to the reduced risk of cardiovascular diseases following moderate red wine drinking [8].

Potential mechanisms of protective effects of beer in cardiovascular system have been studied [119,120,121]. An *in vivo* study reported that light to moderate beer drinking (12.5–25 g/day) could also improve endothelial function through inhibiting vascular oxidative damage and modulating the Akt/endothelial NO synthase pathway, which should be attributed to the non-alcohol components in beer [119]. According to a randomized crossover trial in high cardiovascular male volunteers, the phenolic components in beer (660 mL/day, contained 30 g alcohol for 4 weeks) provided additional protective effects compared to alcohol alone through downregulating the leukocyte adhesion molecules and inflammatory biomarkers in serum [120]. In addition, an intervention study reported that, consumption of non-alcoholic beer (500 mL/day) among post-menopausal women led to reduced oxidative stress which might be beneficial to the cardiovascular system [121].

#### 3.6.2. Potential Mechanisms for Detrimental Effects in the Development of Cardiovascular Disease

Hypertension is the most popular modifiable risk factor of coronary heart disease, heart failure as well as stroke. In alcohol treated rats (percentage of alcohol in drinking water: 5% first week, 10% second week, 20% third to fourth week), a progressive increase of mean arterial pressure was observed through the entire period of study. An upregulation of sympathetic activity was suggested to account for the early stages of hypertension. The idea was proved by increased plasma concentrations of adrenaline and noradrenaline as well as elevated levels of á1-adrenoceptor protein at the first week, and decreased β2-adrenoceptor protein after the second week. However, the maintenance of this hypertensive state might largely depend on the increased secretion of other circulating factors including vasopressin (AVP) and angiotensin II (ANG II). In addition, baroreflex changes since the third week might also play a role in the maintenance as well as cardiac event risks in later stages [122,123].

Most cardiovascular diseases are due to vessel atherosclerosis. In another study, long term alcohol administration (4.5 g/kg/day, equivalent to four standard drinks/day in human) was employed in male Wistar rats to investigate the role of alcohol in the pathogenesis of atherosclerosis. Proinflammatory response, increased cell adhesion molecules and foam cell formation were triggered by alcohol treatment. In addition, the treatment also promoted aortic vascular smooth muscle cell (VSMC) proliferation via oxidized LDL-mediated oxidative stress and homocysteine. All together, these observations might explain the increased atherosclerosis risks among heavy drinkers [124,125]. Vascular calcification is involved in the development of atherosclerosis. Exposure of human vascular smooth muscle cells to alcohol dose dependently increased calcification of extracellular matrix via augmenting Pi-modulated calcification and transition to osteoblast-like cells [126]. In addition, chronic alcoholism increased activity of CYP2E1, leading to oxidative stress and apoptosis, which might ultimately induce tissue remodeling and alcoholic cardiomyopathy [127].

Epidemiological evidence is consistent that alcohol abuse elevates risks of cerebral accidents. In a rat model, short term alcohol treatment enlarged perivascular spaces around small vessels in brain tissue. Besides, the increased synthesize of MMP-2 and MMP-9 induced by alcohol might contribute to the disruption of blood-brain barrier at early stages. On the other hand, pathological changes of basilar arteries were observed after long term treatment of alcohol, as evidenced by exfoliating of endothelial, fragmenting of inner elastic lamina, and thickening of tunica media and adventitia [128].

## 4. Diabetes Mellitus

### 4.1. Evidence from Epidemiological Studies

Researches showed that non-alchohol bioactive compounds from alcoholic beverage consumption could modulate glucose and lipid metabolism, insulin sensitivity, and islet function [129,130]. On the other hand, the significantly varying effects based on amount of drinking, suggested an important role of alcohol itself in the development of diabetes [131]. Evidence suggested that a significant U-shaped association existed between alcohol consumption and diabetes risks. The nadir located at 10–14 g alcohol/day which decreased 18% diabetes risks, and the upper limit for the protection was 63 g/day, whilst above this threshold, there was an approximately liner increase in diabetes risks with increasing alcohol consumption [131]. In addition, beverage type analysis of a prospective study in Greece indicated that these protective effects against long term incidences of diabetes should be mainly attributed to wine and beer [132].

The findings were supported by a randomized controlled trial in Israel. Among well controlled diabetes patients, the initiation of moderate wine consumption (150 mL/day), particularly red wine, was proved to be a safe dietary method for reducing cardiometabolic risks [133]. Besides, a meta-analysis combined results from intervention studies, and reported that moderate drinking in non-diabetic participants might be associated with reduced concentration of fasting insulin and HbA1c [134].

It was suggested that half of all deaths from diabetes could be attributed to cardiovascular incidence, such as stroke and myocardial infarction. Compared with people without diabetes, hazard ratio for diabetes patients was 2.32 (95% CI, 2.11–2.56) for death from cardiovascular causes [135]. According to a prospective study, diabetic patients who reported moderate drinking (≤21 drinks/week for men and ≤14 drinks/week for women) presented decreased risks of cardiovascular accidents (adjusted HR, 0.83; 95% CI, 0.72–0.95; *p* = 0.008), micro vascular complications (adjusted HR, 0.85; 95% CI, 0.73–0.99; *p* = 0.03) as well as all-cause mortality (adjusted HR, 0.87; 95% CI, 0.75–1.00; *p* = 0.05). Furthermore, the beneficial association was especially strong for predominantly wine drinkers. However, researchers also found that heavy drinking (>21 drinks/week in men and >14 drinks/week in women) provided does-dependent increase risks of cardiovascular accidents for diabetes patients (HR 1.02; 95% CI, 1.01–1.04) and all-cause mortality (HR 1.02; 95% CI, 1.00–1.04) [5].

### 4.2. Potential Mechanisms

Several studies found that consumption of moderate alcohol as well as non-alcohol bioactive components from alcoholic beverage could all contribute to the decreased risk of diabetes, but heavy alcohol consumption could increase diabetes risks [129,130,131,136,137].

#### 4.2.1. Potential Mechanisms for Protective Effects in the Development Diabetes Mellitus

Potential mechanisms of alcohol in protection against diabetes have been suggested [138,139,140,141,142,143]. A study reported that alcohol promoted glycolipids uptake as well as enhanced activity of NKT cells, a well-recognized inhibitor in the pathogenesis of type 1 diabetes. The study also postulated that this was the mechanism underlying alcohol reduced diabetes incidences in mice [138]. Besides, in male patients with type 2 diabetes, the insulin sensitivity index increased following intake of 40 g alcohol (40% *v/v*) [139]. Obesity could induce insulin resistance. In obese mice, the treatment of alcohol (20% in drinking water) alleviated insulin resistance by upregulating expression of anti-inflammatory genes [140]. Protective activity of alcohol on insulin sensitivity has been shown to involve increasing adiponectin concentration in both *in vitro* and intervention studies [141,142]. In addition, in male Sprague-Dawley rats, 8 weeks of low dose alcohol administration (2.5%–5% in drinking water) protected against diabetes induced lung injury via enhancing the expression of ALDH2 [143].

Potential mechanisms of wine in protection against diabetes have been widely studied [129,130,144,145]. Peroxisome proliferator-activated receptor γ (PPARγ) plays an important role in glucose and lipid metabolism. Ellagic acid and epicatechin gallate, active components of wine, were reported to have similar affinity to PPARγ of rosiglitazone, which is a standard drug for the treatment of type 2 diabetes. In addition, consumption of 100 mL of the tested red wines provided an activity equivalent about 25%–400% of the daily dose of rosiglitazone [129]. Previous studies have demonstrated that oxidative-nitrative stress, resulting from accumulation of ROS, played a fundamental role in the development of diabetic complications, such as diabetes retinopathy, neuropathy and nephropathy. An *in vitro* study found that the glucose and fructose induced lipid peroxidation in erythrocytes was prevented by wine treatment. Besides, wine treatment promoted glucose uptake and ameliorated fructose triggered osmotic fragility in erythrocytes [130]. Similarly, another study showed that red wine treatment to diabetic rats significantly reduced 3-nitrotyrosine concentration (an indicator of peroxynitrite induced injury) in sciatic nerve, dorsal root ganglia (DRG) neurons, spinal cord, retina and kidney (300 mL/70 kg body weight/day) [144]. These results suggested that the protection against oxidative-nitrative stress might partially explain the decreased diabetes risks associated with wine consumption. The improvement of insulin sensitivity might be implicated in the protection against type 2 diabetes associated with moderate wine drinking. In men with high cardiovascular risks, four weeks of daily intake of red wine or dealcoholized red wine (containing 30 g alcohol) reduced homeostasis model assessment of insulin resistance (HOMA-IR) and mean adjusted plasma insulin, without changing fasting glucose. The results supported a beneficial impact of red wine, particularly the polyphenol components on insulin resistance [145].

Potential mechanisms of beer in protection against diabetes have been also studied [136,137,146]. Xanthohumol is a flavonoid which was reported to exist in hops and beer. Results from an *in vitro* study showed that xanthohumol could decrease the activity of á-glucosidase in a noncompetitive and reversible way via directly binding to the enzyme and triggering conformational alterations. These effects might contribute to the lowered diabetic risks related to beer intake [136]. Type 1 diabetes mellitus is often accompanied by impaired wound healing function due to chronic inflammation and oxidative stress. In a streptozotocin-induced diabetic rat model, 5 weeks of administration of 10 mg/L xanthohumol in drinking water decreased oxidative stress and inflammation, leading to improved wound healing function [137]. Isohumulone is another component in beer, which contributes to the bitterness. It was reported that isohumulone could activate PPAR-á and -γ, and reduce glucose level in plasma. In pre-diabetes subjects, the intake of isohumulone (16–48 mg for 12 weeks) displayed beneficial effects against diabetes as shown by decreased fasting blood glucose and HbA1c [146].

Potential mechanisms of low to moderate drinking in protection against diabetes have been suggested [147,148]. The modulation of metabolism might contribute to the reduced type 2 diabetes risks related to moderate drinking. In patients suffering from diabetes, dyslipidemia is a common complication which is implicated in the vulnerability of these patients to cardiovascular diseases. According to a study, diabetes patients, who reported light to moderate drinking, had reduced lipid-related index, such as the ratio of triglycerides to HDL cholesterol, LDL cholesterol to HDL cholesterol, and the lipid accumulation product [147]. Light-to-moderate drinking (<280 g/week) in healthy women was associated with improved insulin sensitivity, decreased basal insulin secretion and reduced fasting plasma glucagon level [148].

#### 4.2.2. Potential Mechanisms for the Detrimental Effects of Drinking in Diabetes Mellitus

Accumulating evidence has suggested that the impaired glucose tolerance and insulin resistance induced by alcohol contribute to the development of diabetes. A long-term alcohol treatment (5 g/kg/day) to rats enhanced visceral adipose tissue accumulation and impaired glucose tolerance. Further study proposed that the upregulation of inflammatory adipokines (TNF-á, IL-6, leptin and VEGF) and hypoxia indicators (HIF-1á and GLUT1) might be the underlying mechanisms [149]. Another study reported an increase of hepatic expression of gluconeogenic and glycogenolytic enzymes via upregulation of 11β-hydroxysteroid dehydrogenase type 1 and glucocorticoid receptor after alcohol exposure. Studies suggest that these effects were associated with increased glucose concentration, impaired insulin sensitivity and liver damage [150]. Insulin-like growth factor I (IGF-I) and growth hormone (GH), have been proposed to be associated with impaired glucose tolerance in the setting of chronic alcoholism. An *in vivo* study proved this idea by showing increased GH as well as reduced IGF-I induced by alcohol exposure [151]. In addition, periconceptional alcohol intake (12% *v/v* in a liquid diet from 4 days before conception until day 4 of gestation) resulted in offspring with elevated fasting plasma glucose (approximately 10%–25%, *p* < 0.05), impaired glucose tolerance (*p* < 0.05), and decreased insulin sensitivity (*p* < 0.01) at 6 months of age [152].

The dysfunction of pancreatic β-cell was proposed to be another potential mechanism of the positive association between drinking and diabetes. The induction of pancreatic β-cell dysfunction and apoptosis by chronic alcohol administration might act through nitration and downregulation of glucokinase [153]. In a rat model of type 2 diabetes, chronic alcohol consumption (about 36 g/day for 6 weeks) significantly upregulated endoplasmic reticulum stress in pancreas at prediabetes and early stages of diabetes [154]. Fetal alcohol spectrum disorder refers to a variety of adverse developmental outcomes in children caused by alcohol consumption during pregnancy. A study employed alcohol treatment (4 g/kg maternal body weight per day) to pregnant guinea pigs to investigate the effects of chronic prenatal alcohol exposure. Those offspring presented enlarged subcutaneous and visceral adiposity along with increased area of pancreatic adipocyte and reduced area of pancreatic β-cell insulin-like immunopositive, indicating decreased insulin production and/or secretion from in pancreas [155]. In addition, a study used β-cell lines and isolated murine islets to study alcohol influence on insulin production and secretion. Alcohol treatment decreased insulin secretion via inhibiting muscarinic signaling pathway and protein kinase C activation. Besides, alcohol reduced insulin production and increased ER stress in murine islets. The prevention of these deleterious effects by alcohol dehydrogenase inhibitor indicated a key role of alcohol metabolism in this process [156]. Type-A GABA receptor is found in pancreatic β-cell and modulates the autocrine of insulin. Results from a study showed that the alcohol-induced dysfunction of pancreatic β-cell involved inhibition of GABAergic signaling [157].

In a recent study, alcohol (2 g/kg/day) was administrated to diabetic rats for 30 days. Following the treatment, researchers observed significantly increased blood glucose levels and reduced body weight. Besides, the kidney and liver tissues showed histologic damages and increased oxidative stress as assessed by SOD, catalase, and malondialdehyde. These findings indicated that the consumption of alcohol in diabetic rats exacerbated the situation, which might be associated with diabetic complications [158]. In another study, chronic moderate alcohol treatment was administrated to mice from adolescence to adulthood. Following the treatment, researchers observed a dysregulation of type 2 diabetes and insulin signaling pathways in the hypothalamus [159].

## 5. Obesity

### 5.1. Evidences from Epidemiological Studies

Heavy drinking was reported to provide additional risks of obesity, and alcohol-derived calories might play an important role in these increased risks [160]. According to a large scale European study, lifetime heavy alcohol consumption (>90 g/day for male and >60 g/day for female) was positively associated with abdominal obesity in both male and female participants, whereas association between lifetime drinking and general obesity was only observed in male. Besides, beer was suggested to have additional contribution to abdominal adiposity [161]. A meta-analysis supported this opinion. The study suggested a positive association between heavy beer drinking (>500 mL/day) and abdominal obesity [162]. In addition, a cross sectional study found that spirits consumption promoted central and general obesity in men [163]. Therefore, other components in alcoholic beverages might also contribute to the detrimental impact of heavy drinking.

On the other hand, most studies reported protective effects of light to moderate drinking against the development of obesity. For instance, a cross-sectional study among Japanese men found an inverse association between light-moderate alcohol consumption (<44 g/day) and obesity, which was more prominent for younger men than the old [164]. Besides, evidence indicated that alcoholic beverage types modified the protection associated with moderate drinking. According to a prospective cohort study, among normal-weight (BMI 18.5–25 kg/m^2^), postmenopausal women, those who reported moderate drinking had decreased risks of overweight or obese during the 7 years of follow-up. Furthermore, wine intake provided the greatest protection against the development of overweight (HR, 0.75; 95% CI, 0.68–0.84), followed by spirits (HR, 0.85; 95% CI, 0.78–0.93) and beer (HR, 0.90; 95% CI, 0.82–1.00) [165]. It is interesting that this result suggested spirits exerted more protection than beer, but the opinion should be further confirmed. In addition, it is noted that drinking frequency also influences the risks of obesity. According to a cross-sectional study in men aged 50–59 years, after adjusted for confounding factors, for a given total alcohol consumption, drinking frequency was inversely associated with BMI and waist circumference [166].

### 5.2. Potential Mechanisms

#### 5.2.1. Potential Mechanisms for Protective Effects in the Development of Obesity

The protective effects of light to moderate drinking against the development of obesity were mainly attributed to wine and beer polyphenols. Red wine polyphenols were reported to interfere with fat absorption. In rat model, the administration of red wine, at doses equivalent to moderate drinking for human, significantly prevented increase of body weight induced by high-fat diet and the effects were mainly mediated by reducing energy intake. Therefore, modulating of satiety mechanisms might contribute to protection of moderate red wine drinking against obesity [9]. Estrogen could affect lipid accumulation in adipocytes, inhibiting lipogenesis and facilitating lipolysis. Aromatase converts androgen to estrogen. An *in vivo* study reported that red wine administration enhanced expression of aromatase in adipose tissues and decreased size of adipocyte, resulting lower body weight gain compared with the control group [167]. According to another *in vivo* study, dietary xanthohumol supplement (60 mg/kg/day) suppressed the body weight gain of mice, and levels of glucose, total triglycerides, and total cholesterol in plasma were also significantly reduced [168]. Besides, isohumulone from beer could decrease body fat as assessed by BMI in people with prediabetes [140].

#### 5.2.2. Potential Mechanisms for Detrimental Effects in the Development of Obesity

There are several hypotheses regarding the effects of alcohol in obesity development. For instance, alcohol itself could add energy to the meal without decreasing the amount of food intake. Besides, alcohol might amplify appetite in response to stimuli of food. The modulation of satiety could be affected by alcohol either via hormones (e.g., leptin, glucagon-like peptide-1) or central mechanisms (opioid pathway, GABA pathway). In addition, people with alcohol use disorders might also be prone to binge eating [169].

**Figure 1:**
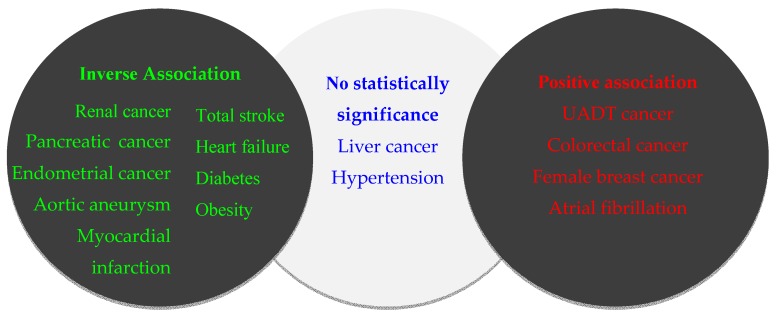
Association between 13.5 g/day of alcohol consumption and disease risks.

The worldwide total *per capita* (15+ years) consumption of pure alcohol was estimated to be 13.5 g/day [1]. The associations between this dose of drinking and risks for selected diseases are set out in Figure 1. This figure is only a rough estimation, because other drinking patterns, especially in case of light to moderate drinking, also play critical roles. In addition, genetic susceptibility, smoking, and other lifestyle factors might modify this association [22,133]. Although there are some limitations in this review, such as the time period, language in the search and selection process could lead to loss of some information, most of important findings published in international journals in recent years have been included.

## 6. Prospects

Although some epidemiological studies suggested the effects of drinking were irrelevant of types of alcoholic beverage, different alcoholic beverages could differ greatly in their composition, which could result in different effects. In the future, alcoholic beverages should be used, rather than alcohol solutions because the results obtained from the alcohol solutions could be different from those from alcoholic beverages, which could contain some other bioactive compounds, such as polyphenols in red wine. Besides, more intervention studies on human are needed to support findings from experimental models with regard to the role of complete red wine in cancer, diabetes and weight control.

## 7. Conclusions

For cancer, alcohol itself and acetaldehyde are Group 1 human carcinogens. Although both *in vitro* and *in vivo* studies have reported the anti-carcinogenic properties of red wine, epidemiological studies could not provide consistent protective effects of light to moderate red wine drinking against carcinogenesis. On the contrary, moderate red wine drinking was suggested to be positively associated with breast cancer risks. For cardiovascular diseases, diabetes and obesity, alcoholic beverages, especially red wine, are protective at light to moderate drinking. It is of note that binge drinking was associated with major cardiovascular accidents, such as myocardial infarction and stroke. For healthy adult drinkers, no more than one drink for females or two drinks for males every day, especially red wine, is acceptable and relatively safe, and might be protective for the cardiovascular system, but occasional binge drinking should be avoided. On the other hand, for non-drinkers, regular moderated drinking, regardless of beverage types, should not be recommended as a way to attain health, because risks for certain diseases, such as colorectal and breast cancer, already increased at light drinking. Furthermore, significantly higher risks for cancer, cardiovascular diseases, diabetes and obesity exist for heavy drinkers and the cession of drinking was suggested to alleviate the conditions.

## Figures and Tables

**Table 1:** Risks of cancer at different sites with alcohol consumption.

Cancer Sites	Relative Risks or Odds Ratio for Drinkers	Reference
Upper aero-digestive tract	less than 12.5 g/day: 1.26 (95% CI, 0.94–1.67);	[17]
	12.6 to 49.9 g/day: 1.79 (95% CI, 1.26–2.53);	
	more than 50 g/day: 3.63 (95% CI, 2.63–5.00)	
Colorectum	less than 12.5 g/day: 1.07 (95% CI, 1.02–1.13);	[4]
	12.6 to 49.9 g/day: 1.23 (95% CI, 1.15–1.32);	
	more than 50 g/day: 1.37 (95% CI, 1.26–1.49)	
Liver	less than 37.5 g/day: 0.91 (95% CI, 0.81–1.02);	[18]
	37.5 g/day or more: 1.16 (95% CI, 1.01–1.34)	
Breast	5 to 15 g/day: 1.06 (95% CI, 1.01–1.11);	[19]
	15 to 30 g/day: 1.12 (95% CI, 1.06–1.19);	
	more than 30 g/day: 1.25 (95% CI, 1.17–1.35)	
Pancreas	less than 37.5 g/day: 0.92 (95% CI, 0.86–0.97);	[20]
	37.5 g/day or more: 1.22 (95% CI, 1.12–1.34)	

*Note*: The drinking amount refers to the dose of pure alcohol. The range of 95% CI below 1 indicates significant protective effects, the range including 1 indicates no statistical significance, and the range above 1 indicates significant harmful effects.
